# Biting off more than you can chew: a rare case of hyperparathyroidism jaw tumour syndrome

**DOI:** 10.1093/omcr/omae162

**Published:** 2025-01-18

**Authors:** Breanna L Hollow, George Chin

**Affiliations:** Medical Registrar and Basic Physician Trainee, Fiona Stanley Hospital, Perth, WA, Australia; Consultant Nephrologist–Department of Nephrology and Transplantation, Fiona Stanley Hospital, Perth, WA, Australia

**Keywords:** hyperparathyroidism jaw tumour syndrome, CDC73, familial hyperparathyroidism

## Abstract

Hyperparathyroidism Jaw Tumour Syndrome (HPT-JT) is a rare autosomal dominant disorder within the familial hyperparathyroidism group. Individuals with the disorder carry a *CDC73* gene mutation that predisposes them to early-onset primary hyperparathyroidism, ossifying jaw tumours, renal cystic disease, uterine tumours and parathyroid carcinomas. We present a case of a 41-year-old man referred to nephrology clinic with haemoproteinuria who was noted to have the constellation of renal cystic disease, personal and family history of hyperparathyroidism and recent jaw tumour excision. Detailed family history prompted whole exome genetic testing which confirmed the presence of a pathogenic *CDC73* gene mutation, ten years after the patient’s initial parathyroidectomy. This case demonstrates the importance of detailed family history taking and the need to consider lesser-known familial hyperparathyroid syndromes to ensure timely diagnosis, genetic testing and cancer surveillance for those affected.

## Introduction

Characterised by hypersecretion of parathyroid hormone (PTH) relative to serum calcium concentration, primary hyperparathyroidism is most commonly caused by sporadic hyperfunctioning parathyroid adenomas, carcinomas or gland hyperplasia [1]. However, the diagnosis of primary hyperparathyroidism in young adults with positive first-degree relatives, should alert discerning clinicians to the possibility of familial hyperparathyroid syndromes. Although conditions such as Multiple Endocrine Neoplasia Type I (MEN1) and IIA (MEN2a) are often discussed [1], the lesser-known Hyperparathyroidism Jaw Tumour Syndrome (HPT-JT) also warrants diagnostic deliberation in the work-up of these familial hyperparathyroid disorders.

## Case report

**Figure 1:**
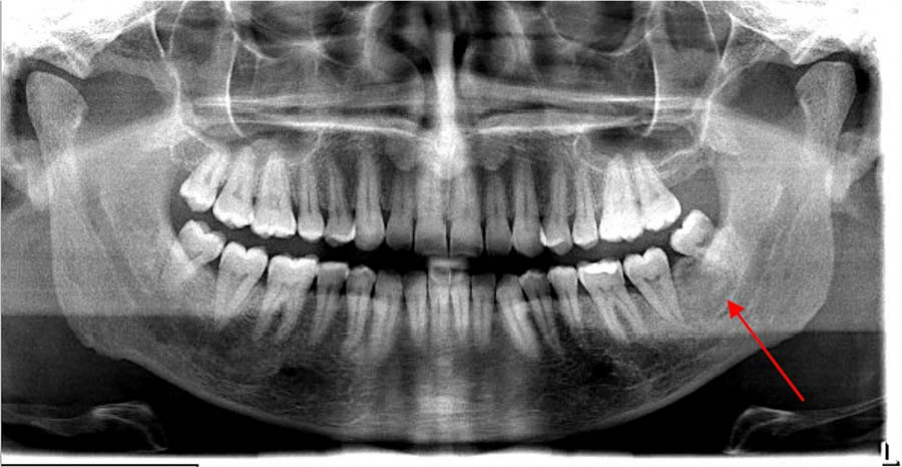
Orthopantomogram demonstrating an ill-defined lesion between the left lower molars (see arrow). This represents an ossifying fibroma.

A 41-year-old man was referred to nephrology for assessment of newly diagnosed painless haemoproteinuria after negative urological assessment. He reported being otherwise well with no recent gastrointestinal or respiratory tract infections. The only recent history of note was that his dentist had excised a benign jaw tumour from his left mandible six weeks earlier (Fig. 1), after he reported increasing asymmetrical jaw swelling. Histopathology confirmed the lesion was an ossifying fibroma.

His medical history included hypertension, alcohol misuse disorder and primary hyperparathyroidism secondary to a unilateral parathyroid adenoma. Our patient had a subtotal parathyroidectomy at the age of 31.

Family history included a similar history of primary hyperparathyroidism in his late father, which was diagnosed at 29 years old. He subsequently died of renal failure at age 67, after he declined dialysis.

On review of our patient, physical examination was unremarkable. Investigations revealed a serum creatinine of 100 umol/L (RR: 60–110), an eGFR of 80 ml/min/1.73m^2^ (RR >60) and a normal adjusted calcium of 2.31 mmol/L (RR: 2.10–2.60). His serum phosphate and parathyroid hormone levels were normal at 0.82 mmol/L (RR: 0.80–1.40) and 6.2 pmol/L (RR: 1.6–9.0) respectively. He was euthyroid with no clinical evidence of hypoadrenalism or hyperadrenalism.

Urinalysis was normal with a urine protein creatinine ratio of 11 mg/mmol. Renal ultrasound revealed multiple simple renal cortical cysts and a non-obstructive left lower pole renal calculus. CT-Intravenous Pyelogram further demonstrated multiple bilateral cortico-medullary cysts and diffuse punctate calcifications of the renal pyramids, consistent with medullary nephrocalcinosis.

This constellation of primary hyperparathyroidism, renal cystic disease and a mandibular ossifying fibroma raised clinical suspicion of Hyperparathyroidism-Jaw Tumour Syndrome (HPT-JT), a rare autosomal dominant condition within the familial hyperparathyroidism group [2]. Our patient was referred for endocrine cancer genetic testing.

Whole exome massively parallel sequencing of the *CDC73* gene confirmed the presence of a heterogenous nonsense variant (c.425C > T; p. Arg 139*). This variant is expected to interrupt the reading frame by creating a premature termination codon, (p. Arg 139*), resulting in loss of function of the tumour suppressor, parafibromin [2]. Lack or down-regulation of parafibromin has been linked to tumorigenesis in head and neck, gastric, lung, colorectal and ovarian cancers, as well as in the pathogenesis of HPT-JT [3].

Our patient’s only living first-degree relative, his brother, was also referred for genetic screening. He was *CDC73* mutation negative and thus, his offspring were not screened.

## Discussion

Previously termed ‘*familial cystic parathyroid adenomatosis,’* HPT-JT predisposes afflicted individuals to ossifying fibromas of the jaw, cystic and neoplastic renal lesions, uterine tumors, parathyroid adenomas and confers an up to 23% lifetime risk of developing a parathyroid carcinoma [2, 4]. First described in 1990 [5], primary hyperparathyroidism remains the most common manifestation of this syndrome, with most *CDC73* mutation carriers developing hyperparathyroidism in late adolescence or early adulthood [4] and an estimated 75%–100% developing hyperparathyroidism by age 70 [2, 4]. Despite this, a significant lag-time between the diagnosis of hyperparathyroidism and HPT-JT is postulated, as occurred here.

To avoid the delayed diagnosis of parathyroid carcinomas, current guidelines for *CDC73* mutation carriers recommend annual neck examination alongside fasting serum calcium, phosphate, vitamin D and parathyroid hormone levels [2]. Routine prophylactic parathyroidectomy is not advised [2]. Five-yearly orthopantomograms and renal ultrasounds are recommended to identify ossifying fibromas and renal cystic disease respectively [2]. There is no current evidence to support routine surveillance for uterine tumours, although early pelvic ultrasound is recommended in symptomatic female carriers [2].

Our patient was diagnosed with primary hyperparathyroidism in his third decade of life. However, the diagnosis of HPT-JT occurred some ten years after his subtotal parathyroidectomy. In a 2021 literature review describing 154 HPT-JT kindreds across 68 articles, the median age at diagnosis was 27 years [4]. All affected individuals had primary hyperparathyroidism (n = 365) with 86.1% of cases occurring secondary to a solitary parathyroid adenoma. Despite the nomenclature, jaw tumours were a less common manifestation of the condition with only 30% of described cases developing fibro-osseous lesions of the mandible or maxilla [4]. Even less common was the presence of renal cystic disease, which was reported in only 15% of cases [4]. Our case, in which all three disease manifestations occurred, exemplifies the heterogenous presentation of this already rare disorder.

Germline inactivating mutations in the tumour suppressor gene *CDC73*, which encodes the 531-amino acid protein parafibromin, are understood to form the genetic basis of this syndrome [4, 6]. More specifically, 75% of HPT-JT patients have a frameshift or nonsense mutation within the coding region of *CDC73* resulting in functional loss of parafibromin via premature protein truncation or nonsense-mediated mRNA decay [4]. The other 25% of afflicted patients have mutations in *CDC73* promotor regions, whole gene deletions or mutations in unidentified genes, that collectively render parafibromin defective [4]. Although definitive genotype–phenotype correlations are yet to be established [4], some studies have suggested that missense mutations are more commonly associated with HPT-JT that lacks typical features (ie isolated primary hyperparathyroidism), whereas mutations that entirely disrupt parafibromin expression present with the classic HPT-JT phenotype encompassing parathyroid, jaw, renal and uterine disease [4, 7, 8]. Our patient’s nonsense mutation rendered parafibromin non-functional, aligning with these hypotheses.

This case aims to remind clinicians to consider familial hyperparathyroid syndromes in patients who have early-onset primary hyperparathyroidism along with positive family histories. Given the heterogeneity of HTP-JT, a high degree of clinical suspicion is required to identify the disorder. Such suspicion is important as timely diagnosis is postulated to improve surveillance and the genetic screening of relatives.

## CRediT statement

GC was responsible for the acquisition of patient data, report editing and review. BH was responsible for collating patient data and drafting the enclosed report. Both authors approved the final report for publication.
